# Critical Analysis of Association Constants between Calixarenes and Nitroaromatic Compounds Obtained by Fluorescence. Implications for Explosives Sensing

**DOI:** 10.3390/molecules28073052

**Published:** 2023-03-29

**Authors:** Alexandre S. Miranda, Paula M. Marcos, José R. Ascenso, Mário N. Berberan-Santos, Peter J. Cragg, Rachel Schurhammer, Christophe Gourlaouen

**Affiliations:** 1 Centro de Química Estrutural, Institute of Molecular Sciences, Faculdade de Ciências, Universidade de Lisboa, Edifício C8, 1749-016 Lisboa, Portugal; 2 IBB-Institute for Bioengineering and Biosciences, Instituto Superior Técnico, Universidade de Lisboa, 1049-001 Lisboa, Portugal; 3 Faculdade de Farmácia da Universidade de Lisboa, Av. Prof. Gama Pinto, 1649-003 Lisboa, Portugal; 4 Centro de Química Estrutural, Institute of Molecular Sciences, Instituto Superior Técnico, Complexo I, Av. Rovisco Pais, 1049-001 Lisboa, Portugal; 5 School of Applied Sciences, Huxley Building, University of Brighton, Brighton BN2 4GJ, UK; 6 Laboratoire de Modélisation et Simulations Moléculaires, Université de Strasbourg, UMR 7140, F-67000 Strasbourg, France; 7 Laboratoire de Chimie Quantique, Université de Strasbourg, UMR 7177, F-67000 Strasbourg, France

**Keywords:** hexahomotrioxacalix[3]arenes, nitroaromatic compounds, calixarene-nitroaromatic supramolecular association, explosives sensing, fluorescence inner filter effect

## Abstract

The binding behaviour of two ureido-hexahomotrioxacalix[3]arene derivatives bearing naphthyl (**1**) and pyrenyl (**2**) fluorogenic units at the lower rim towards selected nitroaromatic compounds (NACs) was evaluated. Their affinity, or lack of it, was determined by UV-Vis absorption, fluorescence and NMR spectroscopy. Different computational methods were also used to further investigate any possible complexation between the calixarenes and the NACs. All the results show no significant interaction between calixarenes **1** and **2** and the NACs in either dichloromethane or acetonitrile solutions. Moreover, the fluorescence quenching observed is only apparent and merely results from the absorption of the NACs at the excitation wavelength (inner filter effect). This evidence is in stark contrast with reports in the literature for similar calixarenes. A naphthyl urea dihomooxacalix[4]arene (**3**) is also subject to the inner filter effect and is shown to form a stable complex with trinitrophenol; however, the equilibrium association constant is greatly overestimated if no correction is applied (9400 M^−1^ vs 3000 M^−1^), again stressing the importance of taking into account the inner filter effect in these systems.

## 1. Introduction

The detection of explosives continues to be an imperative task for all countries in their antiterrorist and homeland security activities. These materials, with their huge destruction potential, can be cheaply and easily prepared. Among them, nitroaromatic compounds (NACs), such as trinitrotoluene (TNT), dinitrotoluene (DNT) and trinitrophenol (TNP, also known as picric acid), are common explosives used for military purposes and the principal components of landmines [1,2]. They are also employed in agrochemical and pharmaceutical industries, being considered environmental pollutants. Concerning human health, they can cause skin irritation, eye cataracts, male infertility, kidney and liver damage, among other diseases [1,2].

Although a wide variety of methods have been used for trace detection of explosives, such as mass spectrometry, ion mobility spectrometry, surface enhanced Raman spectroscopy, gas chromatography coupled with different detectors and nuclear quadruple resonance, expensive and complex equipment is required, causing limitations for their use in real-time applications [3]. Therefore, low-cost detection techniques, with high portability, high sensitivity and selectivity are needed for in-field analyte effective sensing. Among analytical techniques, luminescence-based methods meet these requirements [4].

Lately, a wide range of fluorescence sensors for monitoring explosives in the solid, solution and vapor phases have been developed [5], in particular based on calixarenes [6,7]. Owing to their structural features, these macrocycles have been widely investigated as ion and neutral molecule receptors [8,9]. They possess a pre-organized cavity available in different sizes and conformations, and they can be functionalised at the upper and lower rims, leading to an almost unlimited number of derivatives. Fluorophores such as naphthalene, anthracene and pyrene are among the most incorporated in the calixarene framework, leading to potential fluorescent probes for NACs [10,11,12,13,14,15,16]. The introduction of calix[4]arene moieties in fluorescent conjugated polymers has also been used to detect explosives [17,18].

**Scheme 1:**
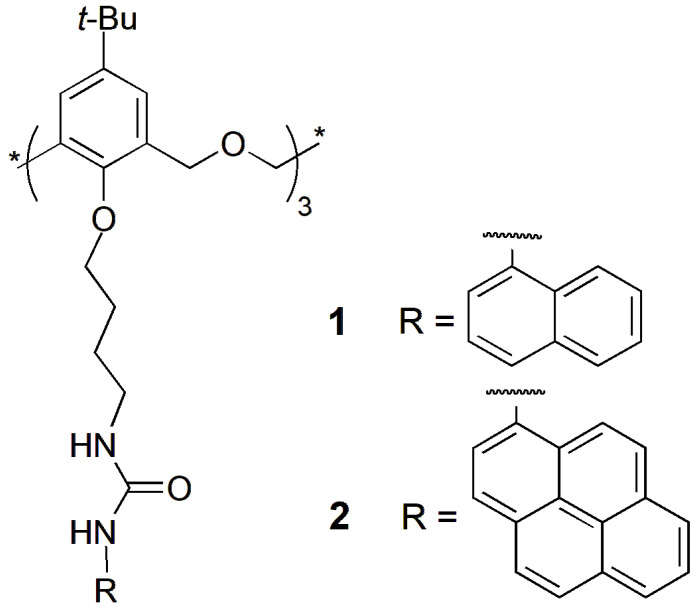
Chemical structures of compounds **1** and **2**.

In the course of our recent studies on anion binding by fluorescent homooxacalixarenes [19,20,21], calixarene analogues in which the CH_2_ bridges are partly or completely replaced by CH_2_OCH_2_ groups [22,23], we have extended our research into the recognition of a different kind of analyte, namely nitroaromatic compounds, by hexahomotrioxacalix[3]arene-based receptors. These macrocyclic compounds, with an 18-membered ring and having only two basic conformations, have already been investigated to detect explosives. A triazole-modified hexahomotrioxacalix[3]arene has been reported as a selective chemosensor for TNP, evidenced by UV-Vis and fluorescence studies [24]. Thus, in this paper, we investigate the binding behaviour of two ureido-hexahomotrioxacalix[3]arene derivatives recently obtained and containing naphthyl (**1**) or pyrenyl (**2**) residues at the lower rim, towards selected NACs (Scheme 1). Their affinity, or lack of it, was determined by UV-Vis absorption, fluorescence and NMR spectroscopy. Different computational methods were also performed to bring further insights to the analysis of the complex formation between the calixarenes and the NACs.

## 2. Results and Discussion

### 2.1. Detection of Nitroaromatic Explosives

#### 2.1.1. UV-Vis Absorption and Fluorescence Studies

**Figure 1:**
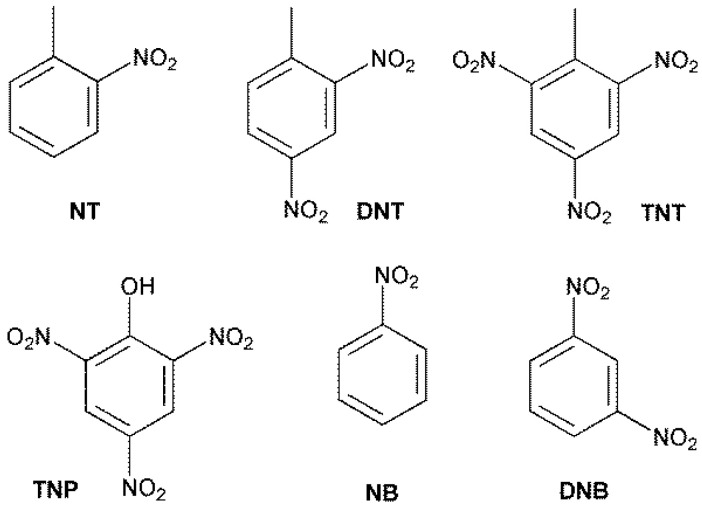
Structures of the nitroaromatic compounds studied.

Recently, we reported the anion binding properties of fluorescent ureido-hexahomotrioxacalix[3]arene receptors **1** and **2**, obtained in the partial cone conformation [21]. Following this line of research, the potential of naphthyl (**1**) and pyrenyl (**2**) ureas as chemosensors to detect nitroaromatic explosives was explored by fluorescence titrations. Selected NACs, 2-nitrotoluene (NT), 2,4-dinitrotoluene (DNT), 2,4,6-trinitrotoluene (TNT), 2,4,6-trinitrophenol (TNP), 1-nitrobenzene (NB) and 1,3-dinitrobenzene (DNB), were tested in dichloromethane and, in the case of **1**, also in acetonitrile (Figure 1). TNT from a military grenade was also analysed, showing similar results to the commercially sourced sample.

**Figure 2:**
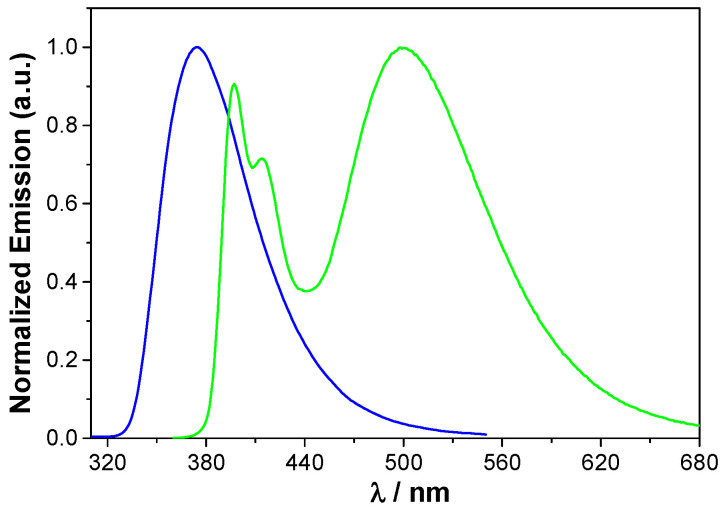
Normalised emission spectra of **1** (blue) and **2** (green) in CH_2_Cl_2_ at 25 °C. [**1**] = 20 μM, $\lambda_{\mathrm{ex}}=$ 300 nm; [**2**] = 10 μM, $\lambda_{\mathrm{ex}}=$ 340 nm.

Compound **1** exhibits emission bands at approximately 375 nm in dichloromethane and acetonitrile, characteristic of the naphthylurea group, while compound **2** presents both monomer (ca. 400 nm) and excimer (ca. 500 nm) bands in dichloromethane arising from the pyrene moieties, as reported previously (Figure 2) [21].

**Figure 3:**
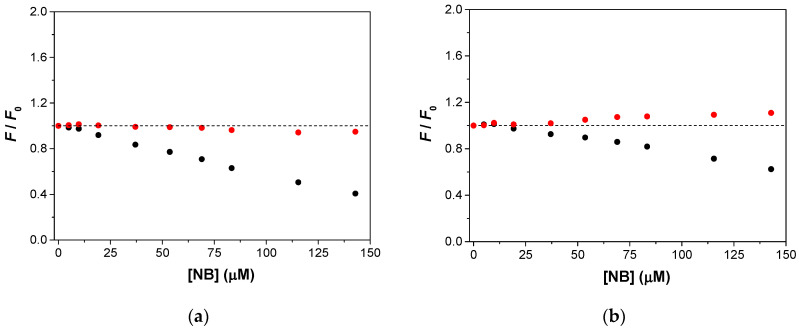
Relative fluorescence intensity (black circles) of Napht urea **1** (20 μM) upon addition of NB (up to 20 equiv) in (**a**) CH_2_Cl_2_ and (**b**) MeCN. *F*_0_ is the intensity in the absence of quencher. Also shown are the intensities corrected for the inner filter effect (red circles). $\lambda_{\mathrm{ex}}=$ 300 nm.

A decrease in the fluorescence intensity was observed upon addition of all the NACs to **1** in both solvents, as shown for NB in Figure 3, as well as upon addition of TNP to **2** in dichloromethane (Figure S1).

**Figure 4:**
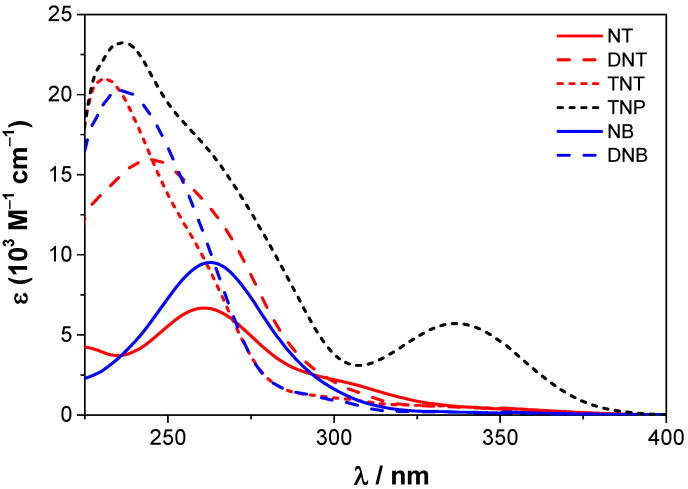
Electronic absorption spectra of the NACs in CH_2_Cl_2_.

As all NAC studied significantly absorb at 300 nm and TNP absorbs also at 340 nm (Figure 4 and Figure S2) the excitation wavelengths used in the case of naphthyl urea **1** and pyrenyl urea **2**, respectively, it is essential to correct for this inner filter effect (absorption of the NAC at the excitation wavelength) before concluding on the existence of genuine static fluorescence quenching (association quenching).

In the case of emission collected only from the centre of the cell, as is the current situation, the correction of the excitation inner filter effect is straightforward, as it immediately follows on from Beer’s law (1):

(1) $$F_{corr}=10^{\Delta A/2}F$$

where *F_corr_* is the corrected fluorescence intensity, *F* the measured fluorescence intensity, and ∆*A* = *A* − *A*_0_, where *A* and *A*_0_ are the absorbances (for 1 cm pathlength) at the excitation wavelength for the solutions of calixarenes **1** and **2** with and without NACs, respectively. The factor of ½ in the exponent accounts for the 0.5 cm effective pathlength of the exciting beam.

**Figure 5:**
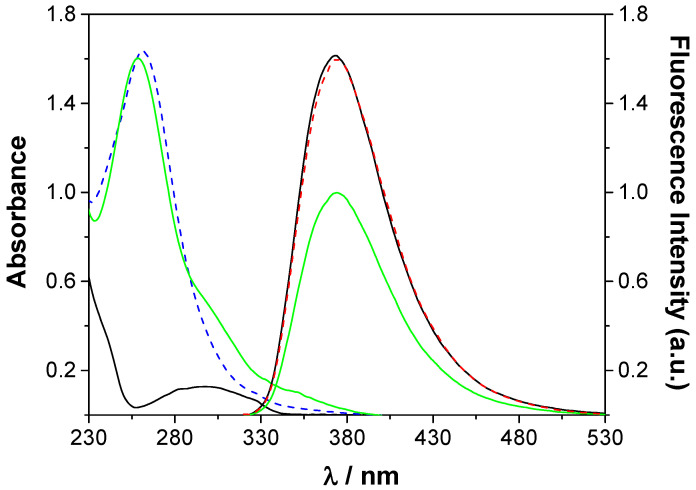
Absorption and fluorescence in CH_2_Cl_2_. Left side (short wavelengths): absorption spectra of **1** (10 μM) alone (black) and in the presence of 20 equiv. of NB (green). The absorption spectrum of 20 equiv. of NB is also shown (dashed blue). Right side (long wavelengths): fluorescence spectra ($\lambda_{\mathrm{ex}}=$ 300 nm) of **1** (10 μM) alone (black) and in the presence of 20 equiv. of NB, before (green) and after (dashed red) correction for the inner filter effect.

As shown in Figure 3 and Figure 5 (see also Figures S3–S7 for the remaining nitroaromatics), it is concluded that the fluorescence quenching by the NACs is only apparent and merely results from the excitation inner filter effect. The apparent quenching indeed correlates with the absorption coefficient of the NAC (Figure 4). No significant association thus exists between calixarenes **1** and **2**, and the NACs studied, both in dichloromethane and acetonitrile.

To further support this conclusion, UV-Vis absorption titrations were carried out with both urea compounds and all the NACs (see, for example, Figure 6 for **1** + DNB and Figures S8–S14), in order to evaluate association constants from absorption data.

The association constants determined (Table 1) for both **1** and **2** are indeed very small, always lower than 0.1 M^−1^, with the exception of **1** + TNP in dichloromethane where it is slightly higher. Thus, the results obtained in all cases indicate only the existence of statistical complexes [25] of **1** and **2** with all NACs studied.

The fluorescence decays of **1** can be fitted with a sum of three exponentials only (Table 2). The complexity of the decay may result from the existence of several different configurations of this supramolecular entity. All three decay components (and hence the average lifetimes) show no significant changes (both in lifetimes and amplitudes) upon NACs addition in substantial amounts (up to 30 equiv.), as shown in Table 2. This invariability, associated with the apparent quenching of the steady-state fluorescence, could mean that static quenching was operative. However, given the negligible association constants found by UV-Vis absorption titrations, the conclusion is simply that no quenching, either static or dynamic, takes place. Indeed, for a possible dynamic quenching mechanism with diffusion control, NAC concentrations (always less than 200 μM) are too low for it to be observable.

The conclusions based on fluorescence are in stark contrast with the claims from two previous reports on NAC sensing using the fluorescence of calixarenes bearing naphthyl [13] and phenylethynylenyl moieties [14]. In these two cases, the excitation inner filter effect is very likely at play but is not mentioned. Both inner filters, excitation and emission, are frequently overlooked or entirely ignored in complexation studies based on fluorescence. Even when not as drastic as in the above-mentioned systems, these effects, if not accounted for, may result in grossly overestimated association constants [18].

**Scheme 2:**
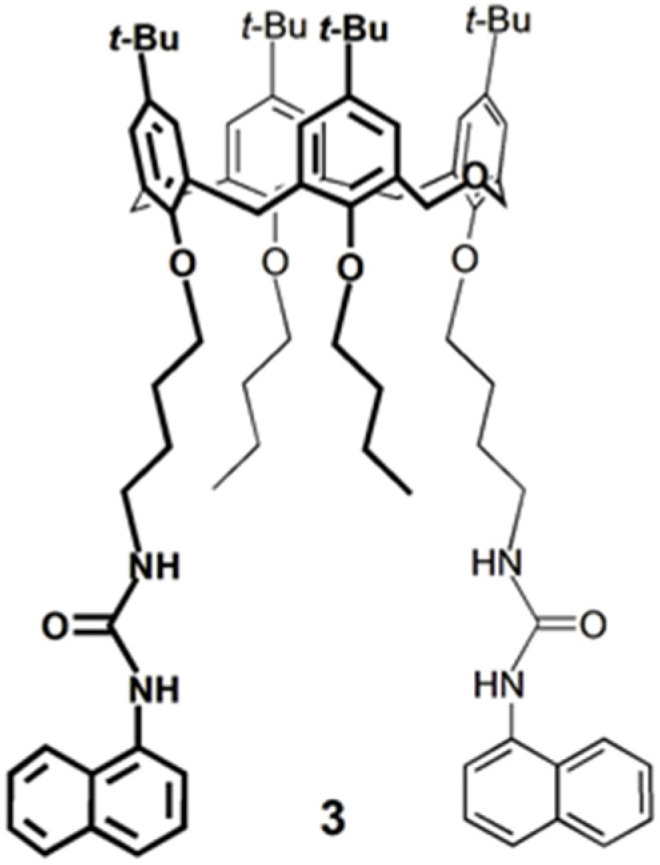
Chemical structure of compound **3**.

**Figure 7:**
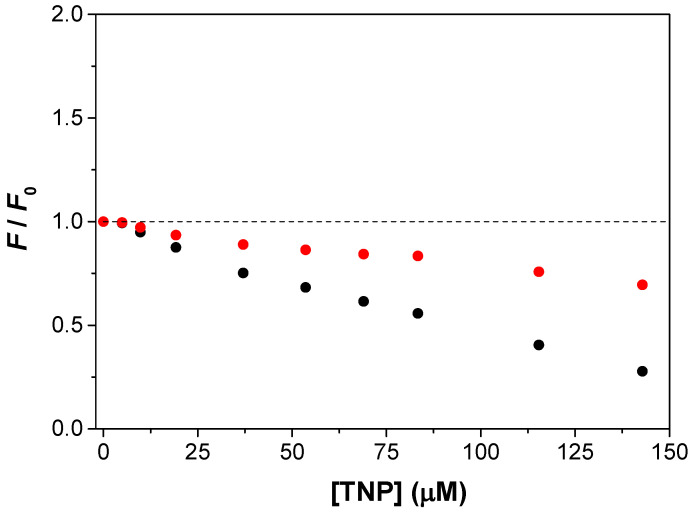
Relative fluorescence intensity (black circles) of Napht urea **3** (10 μM) upon addition of TNP (up to 20 equiv.) in CH_2_Cl_2_. *F*_0_ is the intensity in the absence of quencher. Also shown are the intensities corrected for the inner filter effect (red circles). $\lambda_{\mathrm{ex}}=$ 300 nm.

An example of a homooxacalixarene system where this situation is observed is in the interaction of **3** (Scheme 2) with TNP, with data now presented and analysed. This system also exhibits a strong inner filter effect (Figure S15). As shown in Figure 7, the excitation inner filter correction is not enough to restore the intensities to their original value. It is thus concluded that a genuine quenching exists. Given the concentrations of quencher, dynamic collisional quenching can be ruled out. This is indeed borne out by lifetime measurements as displayed in Table 3. It can thus be concluded that the quenching is static, corresponding to the formation of an association complex.

**Figure 8:**
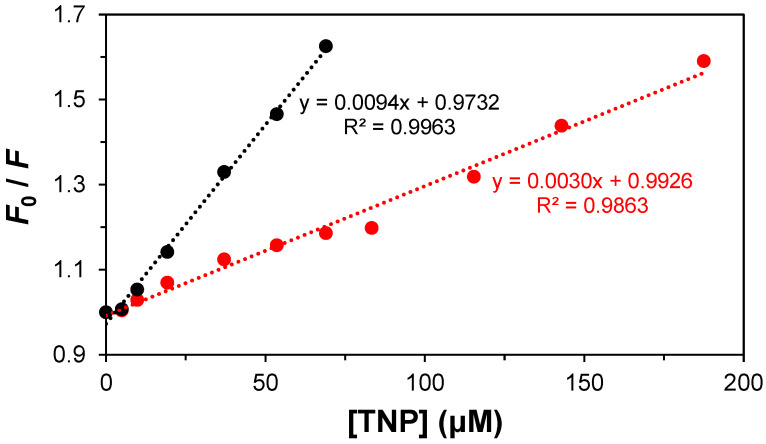
Linear regression of *F*_0_/*F* versus [TNP] (black circles) of Napht urea **3** (10 μM) upon addition of TNP (up to 20 equiv.) in CH_2_Cl_2_. *F*_0_ is the intensity in the absence of quencher. Also shown is the same linear regression for the intensities corrected for the inner filter effect (red circles). $\lambda_{\mathrm{ex}}=$ 300 nm.

Stern-Volmer plots of uncorrected and corrected fluorescence intensity data are shown in Figure 8. It is observed that the uncorrected data deviate from linearity for the higher concentrations, displaying a pronounced upward curvature. Note that, for the sake of clarity in the comparison with corrected data, the last four points are omitted from Figure 8. The slopes are the association constants in μM^−1^. It is thus seen that the uncorrected data yield an apparent association constant of 9400 M^−1^, whereas the corrected data yield an association constant of 3000 M^−1^ for a 1:1 complex.

**Scheme 3:**
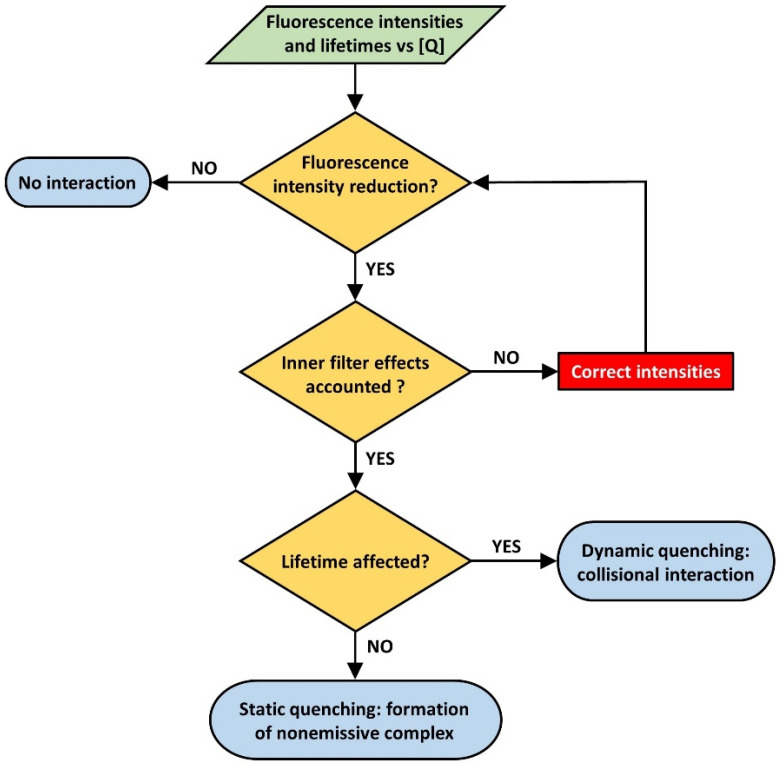
Simplified sequence for the analysis of fluorescence quenching, highlighting the role of potential inner filter corrections. To simplify, only the two main types of quenching are considered, thus discarding situations of mixed quenching.

A sequential approach to the use of fluorescence in quenching studies, namely with NACs, is depicted in Scheme 3, stressing the need to check for potential inner filter effects, both in excitation (absorption by the quencher at the excitation wavelength) and emission (absorption by the quencher at the emission wavelength). We concentrate here on the excitation inner filter effect. In some cases, a change in the excitation wavelength suffices to remove the interference. However, when extensive spectral overlap of the absorption spectra of the fluorescent compound and quencher (NAC) exists, this may not be possible. The use of cells with a small optical path can also work, if enough fluorescence intensity is still obtained, and if the quencher absorption is weak. In any case, a correction should still be applied, as is done here, even if only to confirm that the inner filter effect is indeed negligible. Note that the correction depends on the emitting area sampled by the detector. In many systems, as it happens in this work, only the central part of the cell matters. However, there are correction formulas in the literature that do not apply to this case [18], so users should check the situation for the fluorimeter used, in order not to apply inadequate correction formulas.

#### 2.1.2. NMR Studies

Although unlikely, it is conceivable, in principle, that the host and guest can associate without either the electronic absorption (of both host and guest) or the fluorescence (of the host in this case) being altered, if chromophores and fluorophores are not directly involved in the binding or indirectly affected by it. For this reason, the interaction between ureas **1** and **2** and guests TNP, NB and DNB was also studied by NMR spectroscopy, in an effort to add further insights into the possible binding of the NACs. Proton NMR titrations were performed by adding increasing amounts of the NACs (up to 50 equiv.) to CDCl_3_ solutions of **1** and **2**. Due to the *C*_s_-symmetry of the macrocycles, two sets of naphthyl/pyrenyl aromatic protons are present in the spectra, resulting in extensive peak overlapping and, consequently, greater difficulty to follow them during the titrations. However, it is possible to observe small upfield shift variations (Δ*δ* ≤ 0.06 and 0.15 ppm, respectively, for **1** and **2**) upon addition of TNP to the hosts (see, for example, Figure S16 for **1** + TNP). This shielding effect may suggest weak interactions between the fluorophore moieties and the guest. No variations were seen in the NMR spectra of both ureas throughout the titrations with DNB and NB (see Figure S17 for **1** + DNB).

### 2.2. Computational Studies

#### 2.2.1. Semiempirical Calculations

To further investigate any possible complexation between hexahomotrioxacalix[3]arene ureas **1** and **2** and the NACs, they were subjected to computational analysis. The calixarenes, NAC guests and their complexes were built in Spartan 20 [26] and geometry optimised using molecular mechanics (MMFF) before gas phase semiempirical calculations (PM6) were undertaken using our previously published methods [27,28]. The resulting structures (Figures S18 and S19) indicate the possibility of π–π interactions in some cases, so the thermodynamics of the systems were calculated.

To investigate the stabilities of these complexes, $\Delta G_{\left(\mathrm{binding}\right)}$ was calculated from (2):

(2) $$\Delta G_{\left(\mathrm{binding}\right)}=\Delta G_{\left(\mathrm{complex}\right)}-\left[\Delta G_{\left(\mathrm{host}\right)}+\Delta G_{\left(\mathrm{binding}\right)}\right]$$

An initial investigation, in which the NACs were bound by the macrocycles, generated positive values suggesting that the Gibbs energies of binding were unfavourable. Subsequent calculations, in which the NACs interacted with the macrocycle aromatic substituents, resulted in very negative values (Table S1). While the data support NAC binding, the binding energies are, as is not uncommon with semiempirical calculations, unrealistically large. Consequently, higher level DFT calculations were undertaken.

#### 2.2.2. QM Calculations

The binding of the different guests with naphthyl urea **1** was also studied by a QM/MM approach. The optimisation of the host shows energy minima structures, where the two naphthyl urea arms oriented on the same side of the macrocyclic ring (pair P) interact through π-stacking and hydrogen bonds, while the inverted arm (M) is isolated. In solution, the guests may interact with the inverted arm (M) or with the pair of arms (P). The computed binding energies obtained (Table 4) indicated that the guests form very labile complexes with naphthyl urea **1**, as shown by the positive or almost zero values, except for TNP for which the Gibbs energy suggests some binding.

Further refinement of the structures using DFT methods and analysis of interaction modes between naphthyl urea **1** and a given guest (i.e., DNB) were performed using B3LYP/6-31G(d,p) optimisations in the gas phase, resulting in twenty different initial positions of the guest, leading to fourteen different minimised conformations. Optimised structures and energies of free **1** and DNB ⊂ **1** are given in Figures S20, S21 and Table S2.

The most stable conformation of free **1** possesses intramolecular H-bonds between the two urea groups oriented on the same side of the macrocyclic ring, and it is more stable than the two other minima by about 37–42 kJ·mol^−1^ (Figure S20). The QM optimisations of the DNB ⊂ **1** display a great variety of structures and interaction modes between the calixarene and the guest (Figure S21). All the conformations are within an energy range of 50 kJ·mol^−1^ and show typical host···guest interactions that can be either (i) H-bonding between the oxygen atoms of one or two nitro groups of DNB and the hydrogen atoms from one or two urea groups (see, for example, conformations 10, 8, 7 or 6); (ii) π···π interactions between the aromatic phenyl moiety of DNB and the naphthyl group (conformations 12 or 13); (iii) inclusion of the DNB in the calixarene upper rim (conformations 3 or 4); (iv) CH…π interactions between the aromatic ring of DNB and the urea oxygen (conformations 13 or 14). Complexation energies were calculated taking into consideration the most stable conformation of **1** (conformation 3 in Figure S20), which explains the energy differences obtained for interactions of similar nature, as for example the conformations 10 and 6 which both display a NH···O_NO2_ interaction but differ in the conformation of the calixarene and thus conducted to ΔE of −51.9 vs +3.4 kJ·mol^−1^. These QM calculations clearly show the large variety of loose pairs that can be formed between **1** and DBN molecules.

#### 2.2.3. MD Simulations

**Figure 9:**
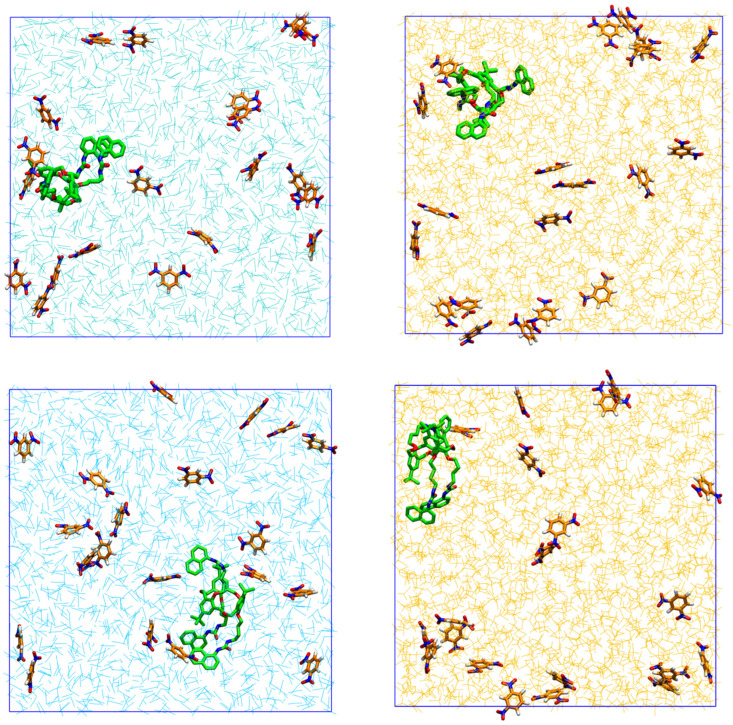
Initial (**top**) and final (**bottom**) snapshots of a box with one calixarene and 20 DNB molecules in acetonitrile (left, in cyan) and in dichloromethane (right, in yellow).

MD simulations were also performed in order to better understand the structure and dynamics of the interactions between naphthyl urea **1** and the guests in solution. Systems containing one host and either TNP, DNB or NT guests were simulated during 50 ns in dichloromethane and acetonitrile solvent. Figure 9 shows initial and final configurations of urea **1** with 20 DNB molecules in both solvents.

**Figure 10:**
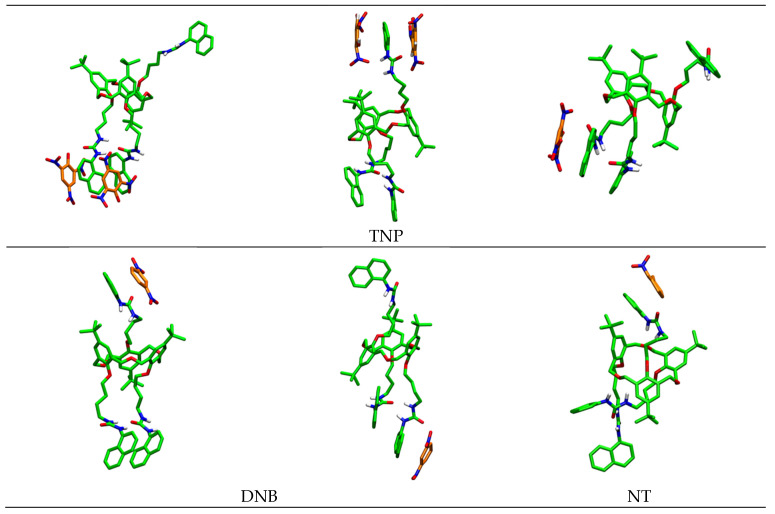
Typical calixarene^…^guest aggregates observed during the simulations (guest in orange).

In solution, **1** always displays intramolecular H-bonds between the two urea arms oriented on the same side of the macrocyclic ring. Meanwhile, the inverted naphthyl urea arm is very flexible and has multiple conformations over time. In this case, as with the two other guests, the DNB molecules are dispersed in the simulation box and display, during the simulation, close contact with the calixarene. In both solvents, free guests remain either isolated or involved in small loose pairs formed via π···π or CH···π interactions. TNP, DNB and NT also interact with the calixarene during the simulation, as illustrated in Figure 10 by snapshots of typical host···guest aggregates. NACs display close contact with the naphthyl groups via π···π bonds, thus forming dimers or trimers with the naphthyl moiety.

In all the simulated systems, the global calixarene···guest interaction energies were evaluated as a function of the nature of both guest and solvent (Table 5). The influence of guest is the same in acetonitrile and dichloromethane. Total interaction energies obtained from three independent MD runs show the same trends with TNP interacting more than 10 times more strongly than NT and 2–3 times than DNB. These variations correlate with the number of urea arm···guest interactions observed during the simulations, which follow the order TNP > DNB > NT in both solutions (Table 5). In acetonitrile, the interactions are quite weak as the average number of urea arm···guest interactions is always below 1 and the lifetimes of the species are around 12–35 ps. Conversely, in dichloromethane, the higher interaction energies are linked to more frequent urea arm···guest contacts with longer lifetimes (up to 110 ps). On average, for all the guests and solvents, MD simulations show the lability and slight interactions between the calixarene and the NACs in direct connection with the experimental results.

These conclusions are also consistent with the QM analysis (see above) and with the value of the calculated electrostatic potential maps (Figure S22) that indicate a better affinity between a naphthyl group, which has roughly a −50 kJ·mol^−1^ potential, and TNP with a highly positive potential (+165 kJ·mol^−1^) compared to DNB (+95 kJ·mol^−1^) and NT (+12 kJ·mol^−1^), thus explaining the large differences observed during the MD simulations.

## 3. Materials and Methods

The naphthyl (**1**) and pyrenyl (**2**) ureido-hexahomotrioxacalix[3]arene compounds studied in this work were previously synthesised [21], as well as the naphthyl urea dihomooxacalix[4]arene **3** [20].

### 3.1. UV-Vis Absorption and Fluorescence Studies

Absorption and fluorescence studies were carried out using a Shimadzu UV-3101PC UV-Vis-NIR spectrophotometer and a Fluorolog F112A fluorimeter in right-angle configuration, respectively. The studies were made in CH_2_Cl_2_ and MeCN at 25 °C. The absorption spectra were recorded between 225 and 400 nm and the emission ones between 320 and 550 nm and using quartz cells with an optical path length of 1 cm. The excitation wavelengths used were at the maximum absorption of the calixarenes, using a right-angle geometry. The titrations were performed cell by cell with different concentrations of nitroaromatic compounds up to 30 equiv. and with a constant concentration of the receptors (10–20 μM). Emission spectra were corrected for the spectral response of the optics and the photomultiplier. In addition, the emission spectra were further corrected for the internal filter effect. The spectral changes were interpreted using the HypSpec 2014 program [29]. Time-resolved fluorescence intensity decays were obtained using the single-photon timing method with laser excitation and microchannel plate detection, with the set-up already described [30]. The excitation wavelength used was at the maximum absorption of the calixarene and the emission wavelength at the maximum emission, using a right-angle geometry. Decay data analysis with a sum of exponentials was achieved by means of a Microsoft Excel spreadsheet specially designed for lifetime analysis that considers the convolution with the IRF.

### 3.2. ^1^H NMR Studies

Several aliquots (up to 50 equiv.) of the nitroaromatic compounds (TNP, NB and DNB) in CDCl_3_ were added to CDCl_3_ solutions of the hosts (1.25 × 10^–3^ M) directly in the NMR tube. The spectra were recorded on a Bruker Avance III 500 spectrometer after each addition of the NAC’s, and the temperature of the NMR probe was kept constant at 25 °C.

### 3.3. Computational Details

#### 3.3.1. QM/MM Calculations

All QM/MM calculations were performed with a Gaussian 09 program [31] using an ONIOM approach [32]. The QM part was carried out at a DFT level of theory with wB97XD functional [33], which includes dispersion corrections. Atoms were described by Pople’s basis set 6-31+G** [34]. The MM part was described by the Universal Force Field UFF [35]. Calculations were performed in solution and the solvent (DCM) described by a PCM [36]. Full geometry optimisations were performed, followed by a frequency calculation. Gibbs free energies were extracted from this frequency calculation.

#### 3.3.2. DFT Calculations

Geometry optimisations were performed with the Gaussian 09 program [31] with the B3LYP density functional [37] and with the 6-31G(d,p) basis set. Starting structures for geometry optimisation were constructed by hand with Spartan [26]. All reported structures were confirmed as energy minima, with no negative eigenvalue in the Hessian matrix. Electrostatic potential maps were performed with Spartan [26]. Details concerning the molecular dynamics simulations are given in the Supporting Information.

## 4. Conclusions

The recognition of several nitroaromatic compounds by two fluorescent ureido-hexahomotrioxacalix[3]arenes bearing naphthyl or pyrenyl residues at the lower rim was investigated by different analytical and theoretical methods. As inferred from electronic absorption, fluorescence, NMR spectroscopy and computational studies, naphthyl urea **1** and pyrenyl urea **2**, both containing aromatic moieties, have no significant interaction with nitroaromatic compounds in both dichloromethane and acetonitrile solutions. DFT calculations performed suggest the formation of very weak and labile complexes between the calixarenes and the NACs, this being corroborated by MD simulations. On the other hand, the observed fluorescence quenching is spurious and can be entirely ascribed to an excitation inner filter effect, whereby the fluorescence decrease results from significant absorption of the excitation radiation by the NAC. This is in stark contrast with the conclusions from some previous reports on NAC sensing using the fluorescence of calixarenes bearing long wavelength absorbing aromatic moieties. A naphthyl urea dihomooxacalix[4]arene (**3**) investigated is also subject to the inner filter effect but forms a stable complex with TNP; however, the equilibrium association constant is largely overestimated (9400 M^−1^ vs 3000 M^−1^) if no correction to the inner filter effect is applied, again stressing the importance of taking into account this effect.

## Figures and Tables

**Figure 6:**
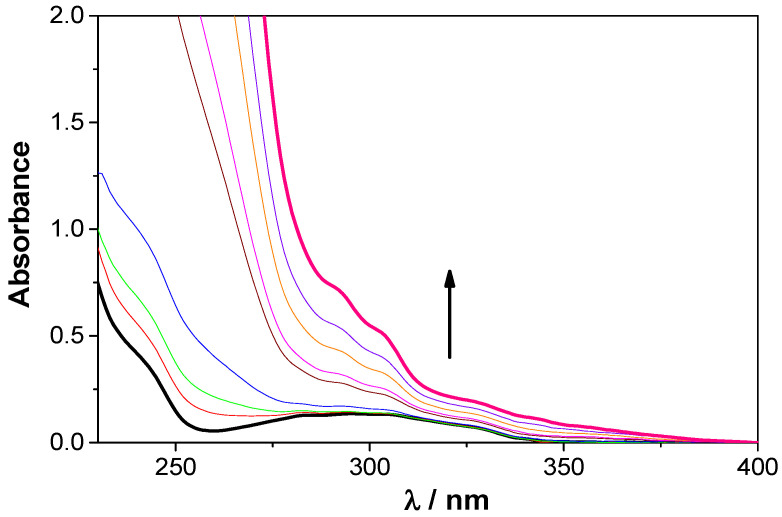
UV absorption spectra of Napht urea **1** (10 μM) upon addition of DNB (up to 30 equiv.) in CH_2_Cl_2_. The arrow indicates increasing amounts of DNB. The spectra of the mixtures are the result of the sum of the two constituent spectra.

**Table 1:** Association constants (*K*_ass_/M^−1^) of ureas **1** and **2** with NACs determined by UV-Vis absorption at 25 °C.

	Solvent	NT	DNT	TNT	TNP	NB	DNB
**1**	CH_2_Cl_2_	<0.1	<0.1	<0.1	0.5	<0.1	<0.1
	MeCN	<0.1	<0.1	<0.1	<0.1	<0.1	<0.1
**2**	CH_2_Cl_2_	<0.1	<0.1	<0.1	<0.1	<0.1	<0.1

**Table 2:** Two- and three-exponential analysis of fluorescence decays of **1** with 30 equiv. of NACs in CH_2_Cl_2_ at 25 °C.

	$\tau_{1}$/ns (%)	$\tau_{2}$/ns (%)	$\tau_{3}$/ns (%)	$\bar{\tau }$/ns
**1** *	0.35 (6)	2.6 (59)	9.3 (35)	4.8
**1** + NT	0.33 (6)	2.5 (58)	8.6 (36)	4.6
**1** + DNT	0.37 (6)	2.6 (59)	9.1 (35)	4.7
**1** + TNT	0.39 (6)	2.7 (60)	9.4 (34)	4.8
**1** + TNP	0.33 (6)	2.5 (57)	8.7 (37)	4.6
**1** + NB	0.35 (6)	2.6 (58)	9.0 (36)	4.7
**1** + DNB	0.35 (6)	2.6 (58)	8.8 (36)	4.7

* $\lambda_{\mathrm{ex}}=$ 300 nm.

**Table 3:** Two- and three-exponential analysis of fluorescence decays of **3** with TNP in CH_2_Cl_2_ at 25 °C.

	$\tau_{1}$/ns (%)	$\tau_{2}$/ns (%)	$\tau_{3}$/ns (%)	$\bar{\tau }$/ns
**3** *	0.26 (10)	2.2 (12)	11.2 (78)	9.0
**3** + 4 equiv. TNP	0.25 (10)	2.0 (12)	10.9 (78)	8.7
**3** + 10 equiv. TNP	0.24 (10)	2.1 (12)	10.8 (77)	8.6

* $\lambda_{\mathrm{ex}}=$ 300 nm.

**Table 4:** QM/MM computed $\Delta G_{\left(\mathrm{binding}\right)}$ (kJ·mol^−1^) for complexes of **1** with NAC guests.

	1	
	M	P
NT	9.2	3.3
DNT	−0.4	3.3
TNT	2.1	1.7
TNP	−1.7	−13.0
NB	12.6	8.8
DNB	9.6	2.5

M denotes inverted urea arm. P denotes pair of urea arms on the same side of the macrocycle.

**Table 5:** Host···guest interaction energies and fluctuations (in kJ·mol^−1^), number of urea arm···guest interaction (N) per arm and fluctuations (arm 1/arm 2/arm 3) and average lifetimes (in ps) ^a^.

		TNP	DNB	NT
MeCN	*E*	−110 ± 75	−29 ± 35	−11 ± 15
		−123 ± 52	−31 ± 31	−13 ± 16
		−123 ± 52	−22 ± 26	−12 ± 16
	N	0.54/0.34/0.82 ± 0.68 (24–35)	0.30/0.26/0.30 ± 0.52 (22–35)	0.21/0.18/0.13 ± 0.42 (14–15)
		0.74/0.41/0.50 ± 0.58 (20–34)	0.32/0.23/0.44 ± 0.47 (20–35)	0.17/0.16/0.17 ± 0.43 (14–15)
		0.30/0.37/0.97 ± 0.53 (24–28)	0.24/0.27/0.17 ± 0.51 (25–30)	0.19/0.17/0.17 ± 0.41 (12–15)
CH_2_Cl_2_	*E*	−246 ± 97	−86 ± 50	−17 ± 20
		−336 ± 60	−59 ± 39	−18 ± 19
		−247 ± 71	−93 ± 61	−35 ± 29
	N	1.98/1.53/1.38 ± 0.68 (50–90)	0.58/0.33/0.83 ± 0.50 (21–37)	0.27/0.21/0.22 ± 0.42 (12–15)
		1.30/1.69/1.79 ± 0.60 (50–110)	0.33/0.51/0.23 ± 0.52 (15–30)	0.20/0.19/0.26 ± 0.40 (14–16)
		1.39/1.10/1.56 ± 0.55 (40–85)	0.43/0.51/0.54 ± 0.45 (28–40)	0.32/0.21/0.27 ± 0.42 (14–16)

^a^ Napht urea **1**···20 molecule guests in MeCN and CH_2_Cl_2_ (3 separate runs).

## Data Availability

Not applicable.

## Supplementary Materials

The following supporting information can be downloaded at https://www.mdpi.com/article/10.3390/molecules28073052/s1, UV-Vis absorption spectra and fluorescence intensity plots; NMR titration spectra; semiempirical calculations; details of molecular dynamics simulations; DFT calculations. References [38,39,40,41,42,43,44] cited in the Supplementary material.

## Sample Availability

Samples of the compounds are not available from the authors.
