# Supplementary material for: Critical Analysis of Association Constants between Calixarenes and Nitroaromatic Compounds Obtained by Fluorescence. Implications for Explosives Sensing
Source: Molecules. 2023 Mar 29;28(7):3052. doi: 10.3390/molecules28073052 (PMC10096452; doi:10.3390/molecules28073052)
Supplement: Supplementary file 1 [file molecules-28-03052-s001.zip › molecules-2290890-supplementary.pdf]

molecules

Supporting Information

Critical Analysis of Association Constants between Calixarenes and Nitroaromatic Compounds Obtained by Fluorescence. Implications for Explosives Sensing

Alexandre S. Miranda [1,2], Paula M. Marcos [1,3,*], José R. Ascenso [4], Mário N. Berberan-Santos [2,*], Peter J. Cragg [5], Rachel Schurhammer [6] and Christophe Gourlaouen [7]

1 Centro de Química Estrutural, Institute of Molecular Sciences, Faculdade de Ciências, Universidade de Lisboa, Edifício C8, 1749-016 Lisboa, Portugal
2 IBB-Institute for Bioengineering and Biosciences, Instituto Superior Técnico, Universidade de Lisboa, 1049-001 Lisboa, Portugal
3 Faculdade de Farmácia da Universidade de Lisboa, Av. Prof. Gama Pinto, 1649-003 Lisboa, Portugal
4 Centro de Química Estrutural, Institute of Molecular Sciences, Instituto Superior Técnico, Complexo I, Av. Rovisco Pais, 1049-001 Lisboa, Portugal
5 School of Applied Sciences, Huxley Building, University of Brighton, Brighton BN2 4GJ, UK
6 Laboratoire de Modélisation et Simulations Moléculaires, Université de Strasbourg, UMR 7140, F-67000 Strasbourg, France
7 Laboratoire de Chimie Quantique, Université de Strasbourg, UMR 7177, F-67000 Strasbourg, France

* Correspondence: pmmarcos@fc.ul.pt (P.M.M.); berberan@tecnico.ulisboa.pt (M.N.B.-S.)

List of contents

1. UV-Vis absorption spectra and fluorescence intensity plots…………..………….. 2
2. RMN titration spectra……………………………………...………………….. 9
3. Semiempirical calculations……………………………………………………...11
4. Details of molecular dynamics simulations………………..………………………12
5. DFT calculations 14

UV-Vis absorption spectra and fluorescence intensity plots

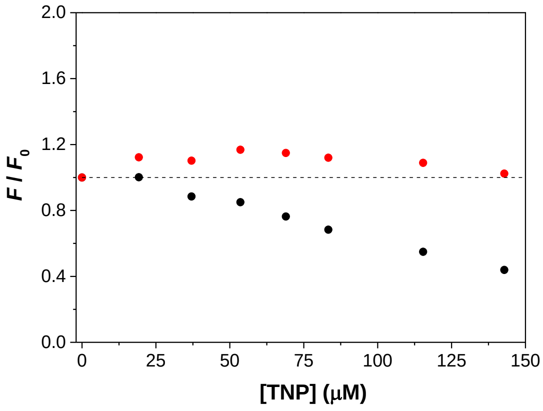

Figure S1. Corrected (red circles) and uncorrected (black circles) F / F_0 versus [TNP] in CH_2Cl_2, F refers to the values of fluorescence intensity of Pyr urea **2** upon addition of TNP and F_0 is the value of fluorescence intensity of Pyr urea **2** (always 20 μM).

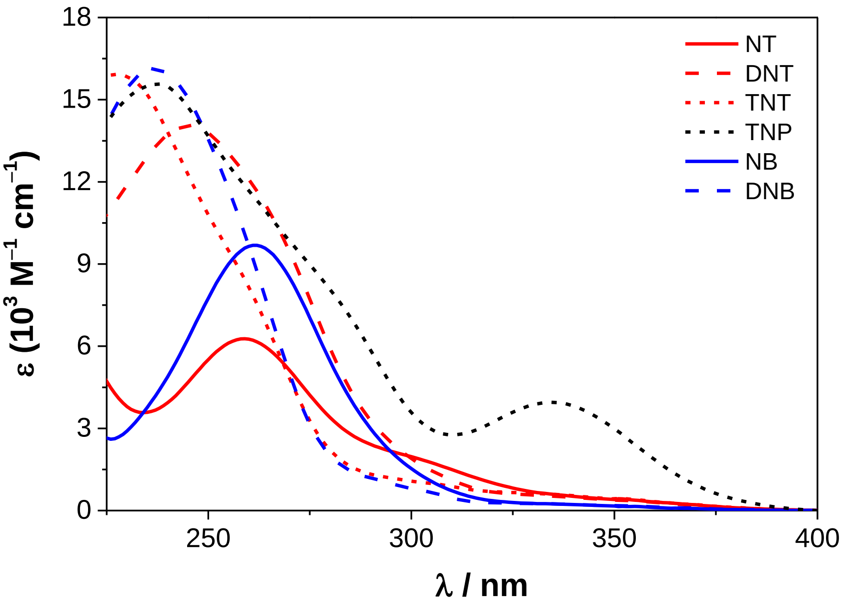

Figure S2. Molar absorption coefficients of nitroaromatic compounds (NT, DNT, TNT, TNP, NB and DNB) in MeCN.

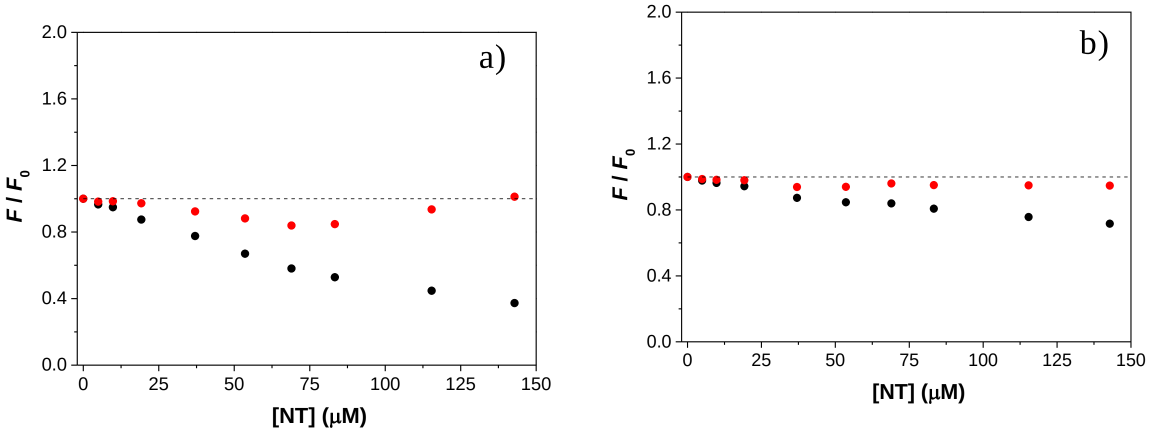

Figure S3. Corrected (red circles) and uncorrected (black circles) F / F_0 versus [NT] in a) CH_2Cl_2 and b) MeCN, F refers to the values of fluorescence intensity of Napht urea **1** upon addition of NT and F_0 is the value of fluorescence intensity of Napht urea **1** (always 20 μM).

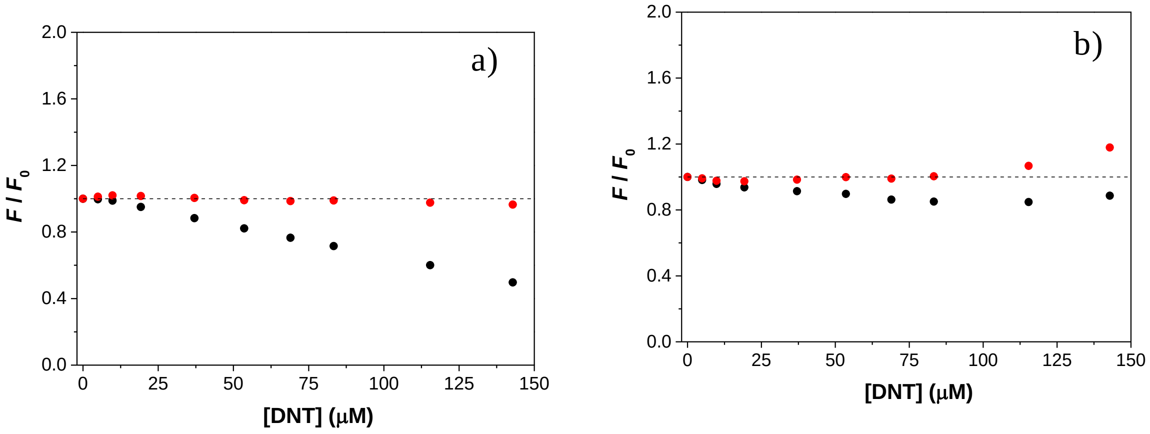

Figure S4. Corrected (red circles) and uncorrected (black circles) F / F_0 versus [DNT] in a) CH_2Cl_2 and b) MeCN, F refers to the values of fluorescence intensity of Napht urea **1** upon addition of DNT and F_0 is the value of fluorescence intensity of Napht urea **1** (always 20 μM).

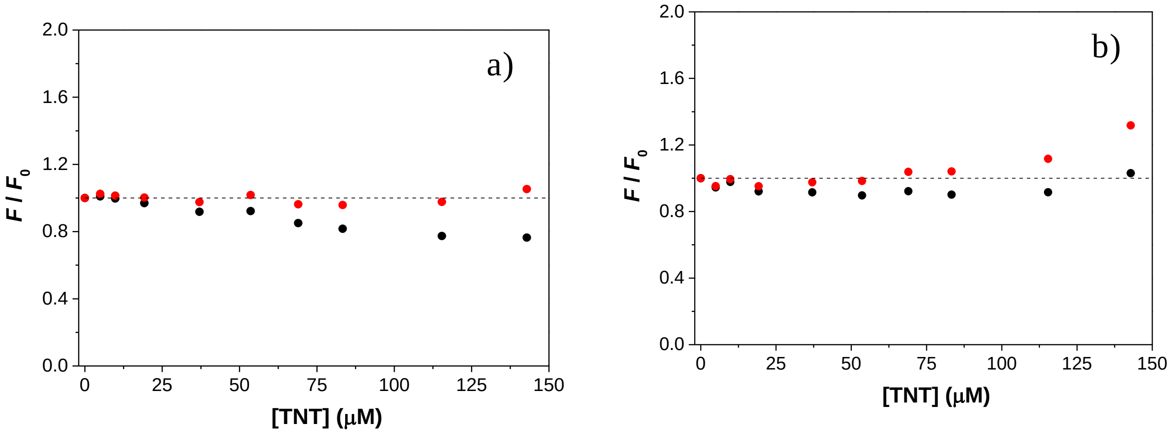

Figure S5. Corrected (red circles) and uncorrected (black circles) F / F_0 versus [TNT] in a) CH_2Cl_2 and b) MeCN, F refers to the values of fluorescence intensity of Napht urea **1** upon addition of TNT and F_0 is the value of fluorescence intensity of Napht urea **1** (always 20 μM).

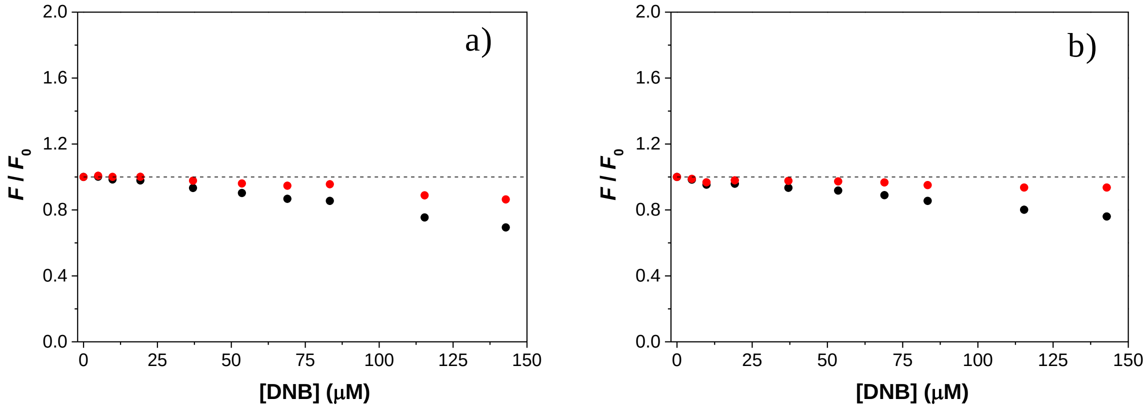

Figure S6. Corrected (red circles) and uncorrected (black circles) F / F_0 versus [DNB] in a) CH_2Cl_2 and b) MeCN, F refers to the values of fluorescence intensity of Napht urea **1** upon addition of DNB and F_0 is the value of fluorescence intensity of Napht urea **1** (always 20 μM).

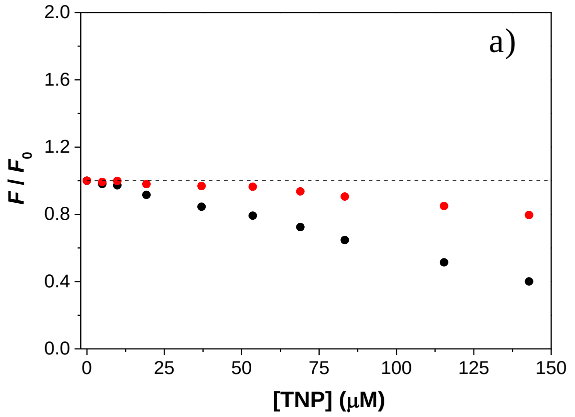

Figure S7. Corrected (red circles) and uncorrected (black circles) F / F_0 versus [TNP] in a) CH_2Cl_2 and b) MeCN, F refers to the values of fluorescence intensity of Napht urea **1** upon addition of TNP and F_0 is the value of fluorescence intensity of Napht urea **1** (always 20 µM).

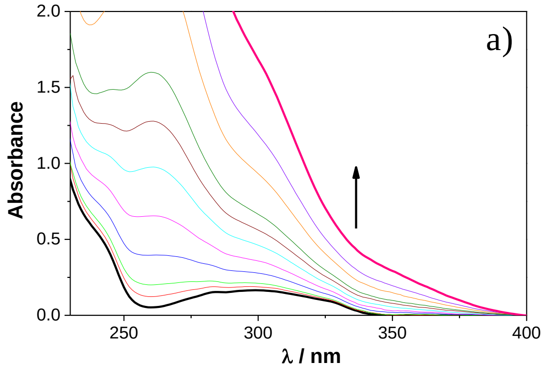

Figure S8. UV absorption spectra of Napht urea **1** (10 µM) upon addition of NT (up to 30 equiv.) in (a) CH_2Cl_2 and (b) MeCN. The arrow indicates increasing amounts of NT. The spectra of the mixtures are the result of the sum of the two constituent spectra.

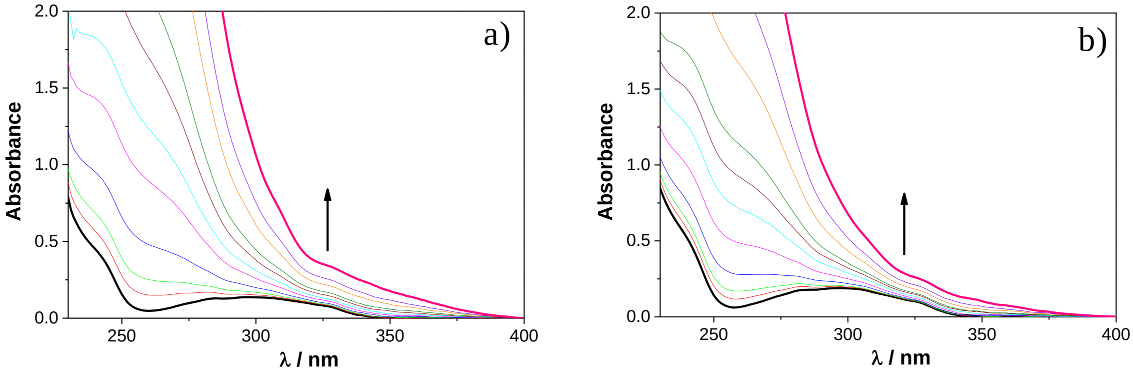

Figure S9. UV absorption spectra of Napht urea **1** (10 μM) upon addition of DNT (up to 30 equiv.) in (a) CH_2Cl_2 and (b) MeCN. The arrow indicates increasing amounts of DNT. The spectra of the mixtures are the result of the sum of the two constituent spectra.

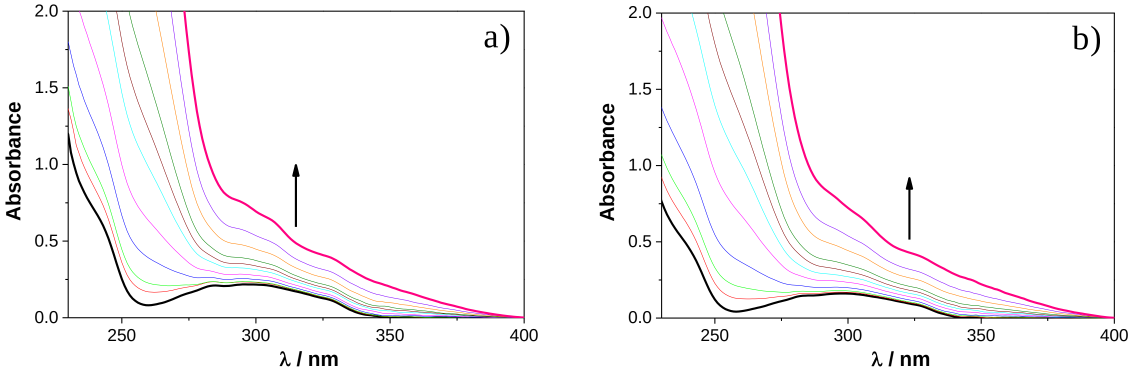

Figure S10. UV absorption spectra of Napht urea **1** (10 μM) upon addition of TNT (up to 30 equiv.) in (a) CH_2Cl_2 and (b) MeCN. The arrow indicates increasing amounts of TNT. The spectra of the mixtures are the result of the sum of the two constituent spectra.

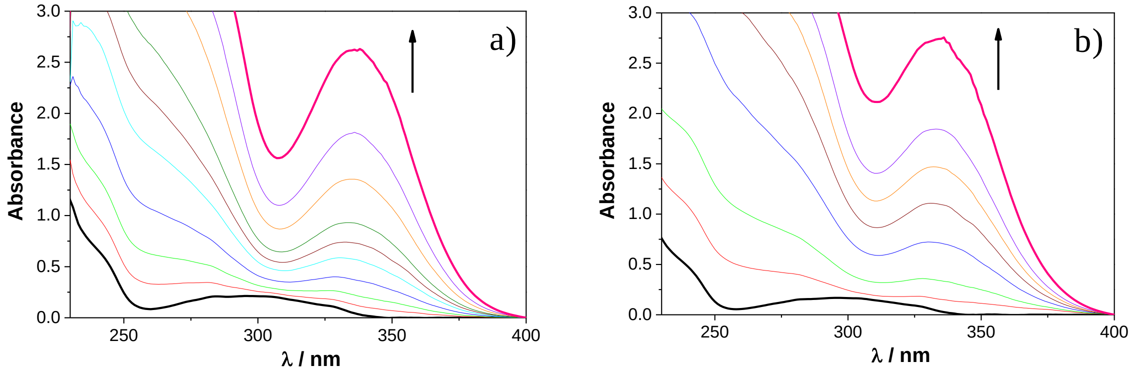

Figure S11. UV absorption spectra of Napht urea **1** (10 μM) upon addition of TNP (up to 30 equiv.) in (a) CH_2Cl_2 and (b) MeCN. The arrow indicates increasing amounts of TNP. The spectra of the mixtures are the result of the sum of the two constituent spectra.

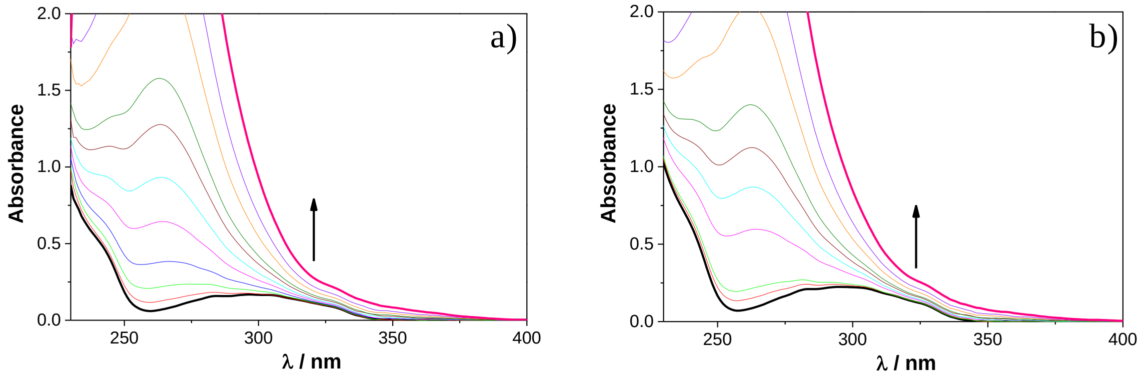

Figure S12. UV absorption spectra of Napht urea **1** (10 μM) upon addition of NB (up to 30 equiv.) in (a) CH_2Cl_2 and (b) MeCN. The arrow indicates increasing amounts of NB. The spectra of the mixtures are the result of the sum of the two constituent spectra.

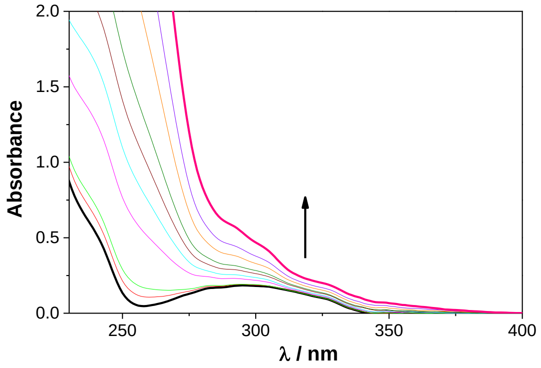

Figure S13. UV absorption spectra of Napht urea **1** (10 μM) upon addition of DNB (up to 30 equiv.) in MeCN. The arrow indicates increasing amounts of DNB. The spectra of the mixtures are the result of the sum of the two constituent spectra.

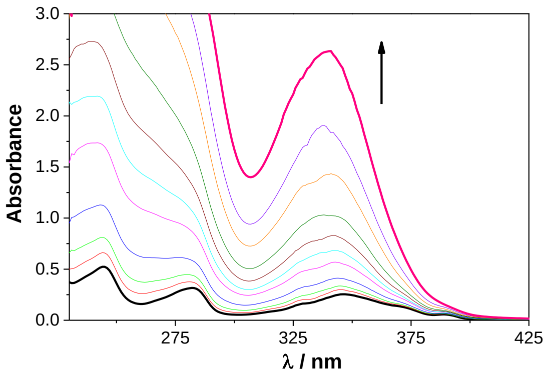

Figure S14. UV absorption spectra of Pyr urea **2** (10 μM) upon addition of TNP (up to 30 equiv.) in CH_2Cl_2. The arrow indicates increasing amounts of TNP. The spectra of the mixtures are the result of the sum of the two constituent spectra.

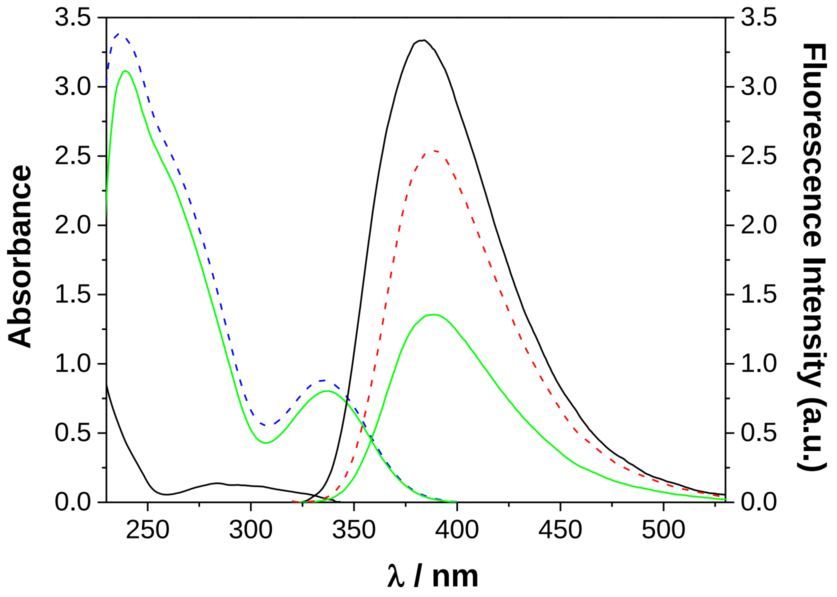

Figure S15. Absorption and fluorescence in CH_2Cl_2. Left side (short wavelengths): absorption spectra of **3** (10 μM) alone (black) and in the presence of 15 equiv of TNP (green). The absorption spectrum of 15 equiv of TNP is also shown (dashed blue). Right side (long wavelengths): fluorescence spectra (λ_{ex} = 300 nm) of **3** (10 μM) alone (black) and in the presence of 15 equiv of TNP, before (green) and after (dashed red) correction for the inner filter effect.

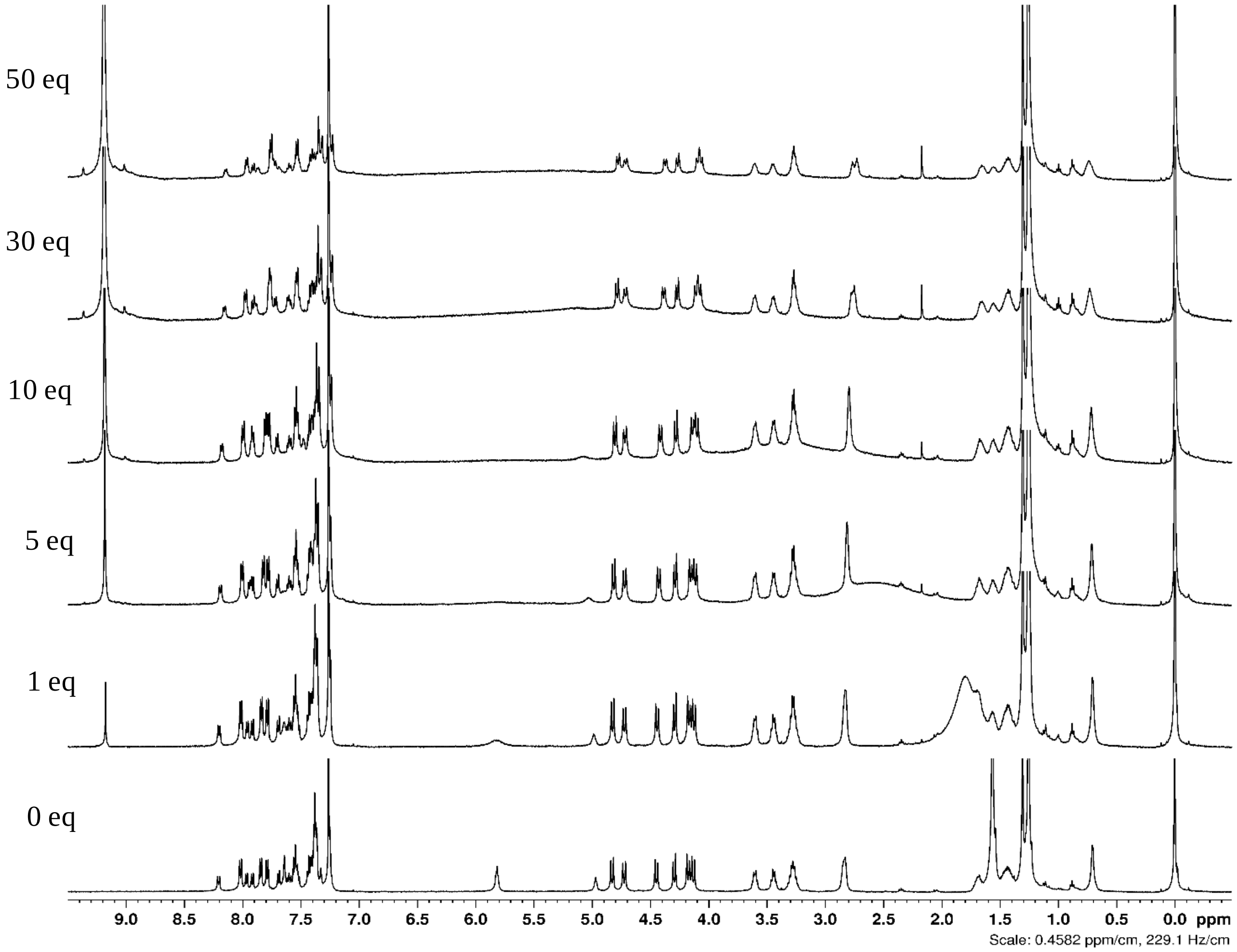

Figure S16. ^{1}H NMR spectra (500 MHz, $CDCl_3$, 25 °C) of naphthyl urea **1** with several equiv of TNP.

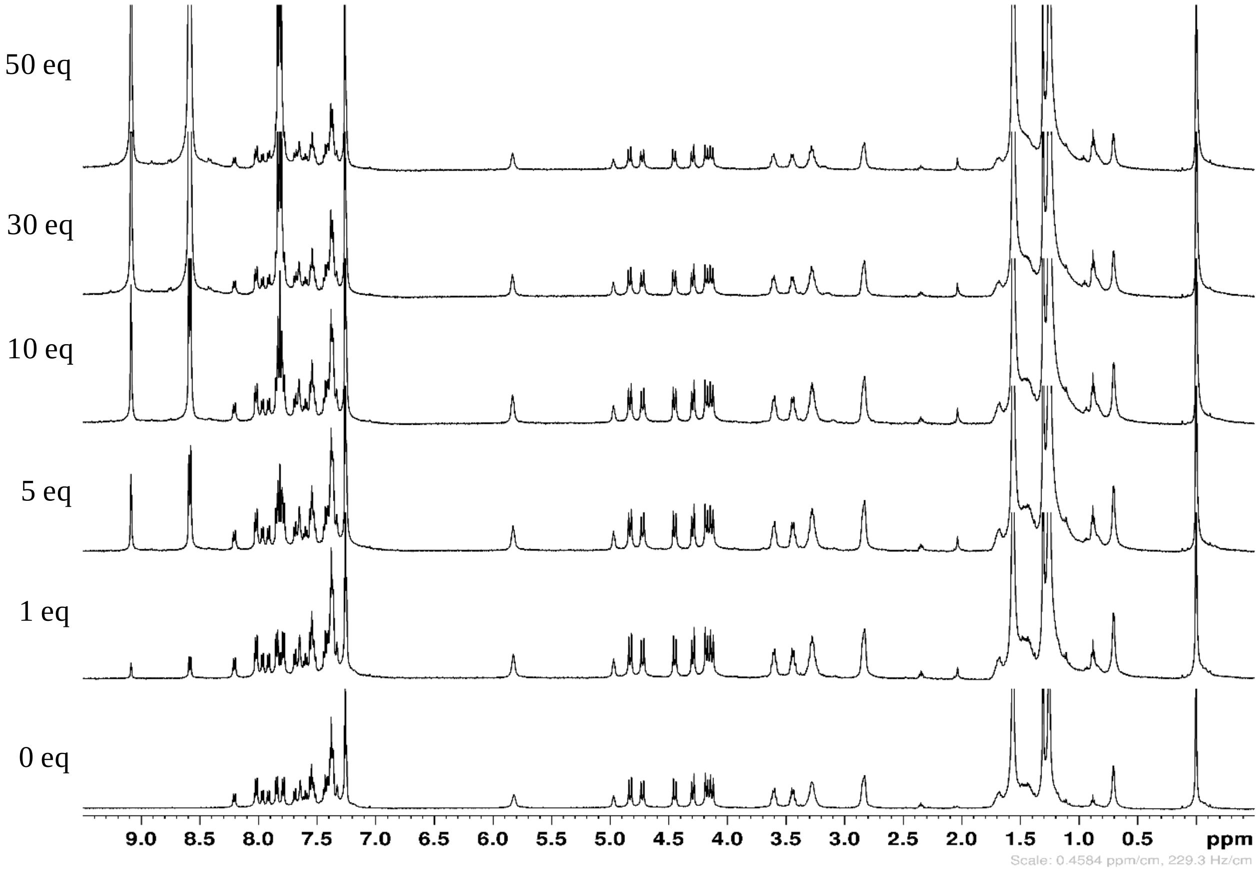

Figure S17. ^1H NMR spectra (500 MHz, $CDCl_3$, 25 °C) of naphthyl urea **1** with several equiv of DNB.

Semiempirical calculations

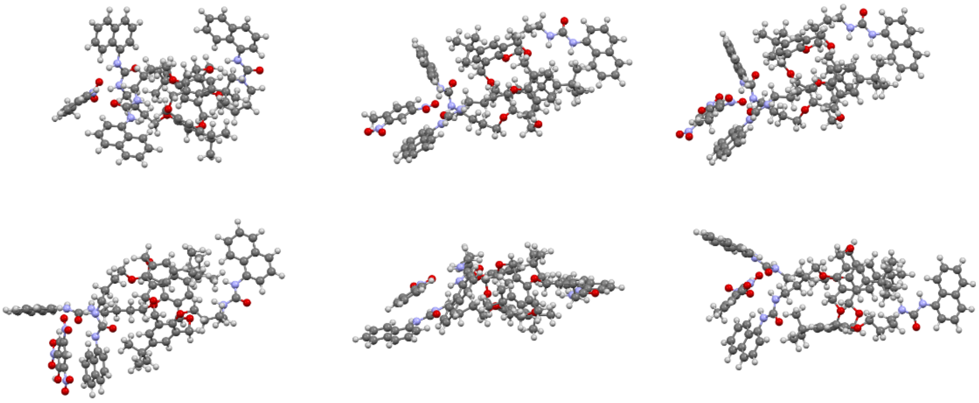

Figure S18. Simulated complexes of **1** with: (top left to right) NT, DNT, TNT; (bottom left to right) TNP, NB and DNB.

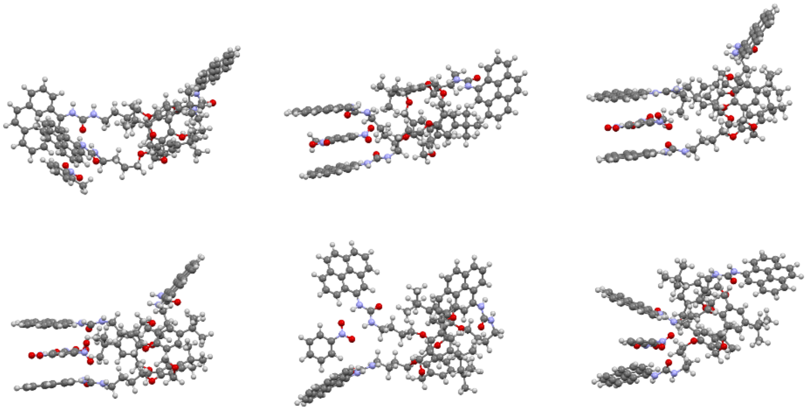

Figure S19. Simulated complexes of **2** with: (top left to right): NT, DNT, TNT; (bottom left to right) TNP, NB and DNB.

Table S1. Calculated $G_{(binding)}$ (kJ mol^{-1}) for complexes of **1** and **2** with NAC guests

	NT	DNT	TNT	TNP	NB	DNB
**1**	−1136.1	−1101.7	−1088.9	−1252.8	−1056.7	−1050.1
**2**	−789.8	−854.9	−802.5	−962.8	−778.5	−768.7

Classical molecular dynamics simulations

The different systems were simulated by classical molecular dynamics (MD) using the AMBER.18 GPU software [38] in which the potential energy U (Eq. 1) is empirically described by a sum of bond, angle and dihedral deformation energies and a pair wise additive 1-6-12 (electrostatic + van der Waals) interactions between non-bonded atoms.

Eq 1

$$U = \sum_{bonds} k_b(r - r_0)^2 + \sum_{angles} k_\theta(\theta - \theta_0)^2 + \sum_{dihedrals} V_n[1 + cos(n\phi - \gamma)] + \sum_{i=1}^{N-1} \sum_{j=i+1}^{N} \left[\frac{A_{ij}}{R_{ij}^{12}} - \frac{B_{ij}}{R_{ij}^{6}} + \frac{q_i q_j}{\varepsilon_0 r_{i,j}} \right]$$

Each simulated system is composed of one calixarene and 20 free guest molecules (DNB, NT or TNP) solvated either in a CH_2Cl_2 containing 1500 molecules or in an acetonitrile box with 2000 molecules (box size is around 60 × 60 × 60^3). The calixarene and the nitroaromatic compounds are initially randomly dispersed in the simulation box. For each case (couple of ligand and solvent) three initial conformations were simulated independently and lead to the same conclusions.

Force field parameters from the GAFF force field [39] and atomic charges were obtained using the RESP methodology [40]. For CH_2Cl_2 it was used the model of Kollmann et al. [41] and the united atom model for acetonitrile [42]. Cross terms in van der Waals interactions were constructed using the Lorentz-Berthelot rules. 1-4 van der Waals and 1-4 electrostatic interactions were scaled by a factor of 1.2 and 2, respectively. The MD simulations were performed at 298.15 K starting with random velocities. All simulations have been carried out using 3D Periodic boundary conditions. An atom-based cut-off of 12 Å for nonbonded interactions was applied and long-range electrostatics were calculated using the

Ewald summation method in the particle mesh Ewald (PME) approximation, while a long-range correction for vdW interaction was applied [43].

Different systems were first equilibrated by 0.3+0.3+0.3 ns of dynamics in the NPT ensemble at respectively 25 K, 100 K and 300 K, followed by 0.3 ns of dynamics in the NVT ensemble. Finally, production runs of at least 50 ns in the NVT ensemble were then simulated. The system temperature was controlled using a Berendsen thermostat with a time step of 1 ps. A time step of 2 fs was used to integrate the equations of motion via the Verlet leapfrog algorithm. Trajectories were saved every 1 ps. Snapshots along the trajectory were taken using the VMD software [44].

38. Case, D.A.; Ben-Shalom, I.Y.; Brozell, S.R.; Cerutti, D.S.; Cheatham, E.; Cruzeiro, V.W.D.; Darden, T.A.; Duke, R.E.; Ghoreishi, D.; Gilson, M.K.; et al. *AMBER 18*, University of California, San Francisco, CA, USA: 2019.
39. Wang, J.; Wolf, R.M.; Caldwell, J.W.; Kollman, P.A. Development and testing of a general amber force field. *J. Comput. Chem.* **2004**, *25*, 1157–1174.
40. Bayly, C.I.; Cieplak, P.; Cornell, W.D.; Kollman, P.A. A well-behaved electrostatic potential based method using charge restraints for deriving atomic charges: the RESP model. *J. Phys. Chem.* **1993**, *97*, 10269–10280.
41. Fox, T.; Kollman, P.A. Application of the RESP methodology in the parametrization of organic solvents. *J. Phys. Chem. B* **1998**, *102*, 8070–8079.
42. Jorgensen, W.L.; Briggs, J.M. Monte Carlo simulations of liquid acetonitrile with a three-site model. *Mol. Phys.* **1988**, *63*, 547–558.
43. Allen, M.P.; Tildesley, D.J. *Computer simulation of liquids*, Clarendon Press: Oxford, UK, 1987.
44. Humphrey, W.; Dalke, A.; Schulten, K. VMD: visual molecular dynamics. *J. Mol. Graph.* **1996**, *14*, 33–38.

DFT calculations

Conformation 1<br><br>–4150.1818 h<br>*ΔE*= +40.5 kJ.mol^{-1}	Conformation 2<br><br>–4150.1834 h<br>*ΔE*= +35.9 kJ.mol^{-1}	Conformation 3<br><br>–4150.1972 h<br>*ΔE*= 0.0 kJ.mol^{-1}
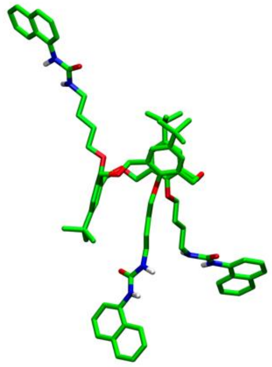	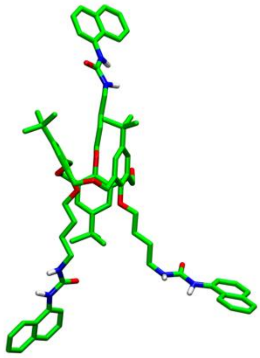	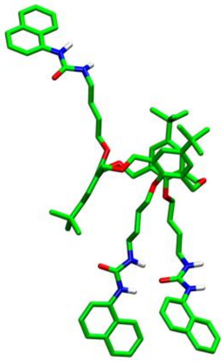

Figure S20. Final structures of Napht urea **1** minimum conformations obtained by B3LYP/6-31G(d,p) QM optimizations. Total energy *E* in hartrees and energy variation between the current and the most stable conformer (in kJ mol^{-1}).

Conf 1 / *ΔE* = –4.6

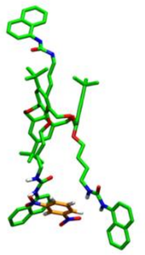

Conf 2 / *ΔE* = 1.2

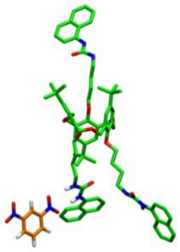

Conf 3 / *ΔE* = –3.8

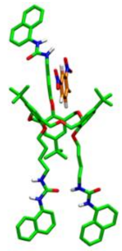

Conf 4 / *ΔE* = –4.6

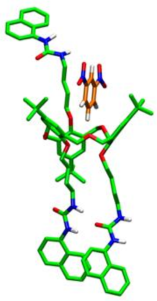

Conf 5 / *ΔE* = –28.8

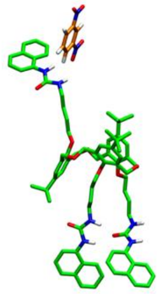

Conf 6 / *ΔE* = 3.3

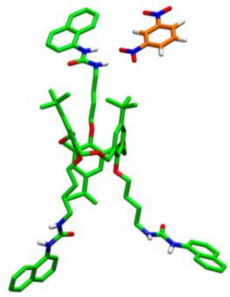

Conf 7 / *ΔE* = –9.6

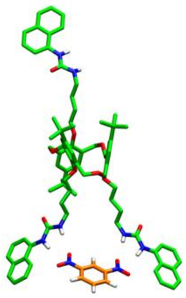

Conf 8 / *ΔE* = –39.3

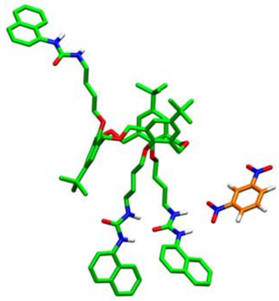

Conf 9 / *ΔE* = –30.7

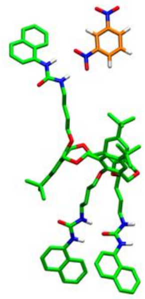

Conf 10 / *ΔE* = –51.8

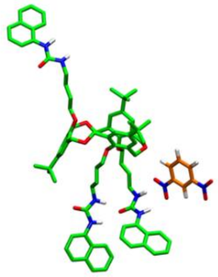

Conf 11 / *ΔE* = –35.4

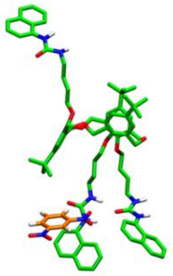

Conf 12 / *ΔE* = –30.4

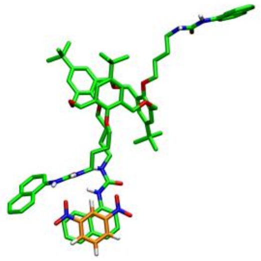

Conf 13 / *ΔE* = –8.1

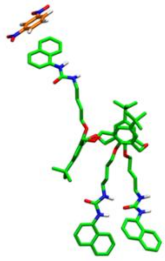

Conf 14 / *ΔE* = –22.3

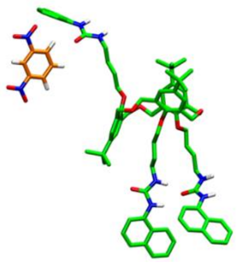

Figure S21. Final structures of DNB ⊂ **1** minimum conformations obtained by B3LYP/6-31G(d,p) QM optimizations. Interaction energy Δ*E* in kJ mol^{-1}.

Table S2. Total and DNB complexation energies obtained by B3LYP/6-31G(d,p) QM optimizations (free Napht urea **1** total energy is 4150.1972 h)

Conformation	Total energy (in hartree)	Complexation energy (in kJ mol^{-1})
1	–4791.4515	–4.5
2	–4791.4493	1.3
3	–4791.4512	–3.7
4	–4791.4515	–4.5
5	–4791.4608	–28.9
6	–4791.4485	3.4
7	–4791.4534	–9.4
8	–4791.4648	–39.3
9	–4791.4615	–30.7
10	–4791.4696	–51.9
11	–4791.4633	–35.4
12	–4791.4614	–30.4
13	–4791.4529	–8.1
14	–4791.4583	–22.3

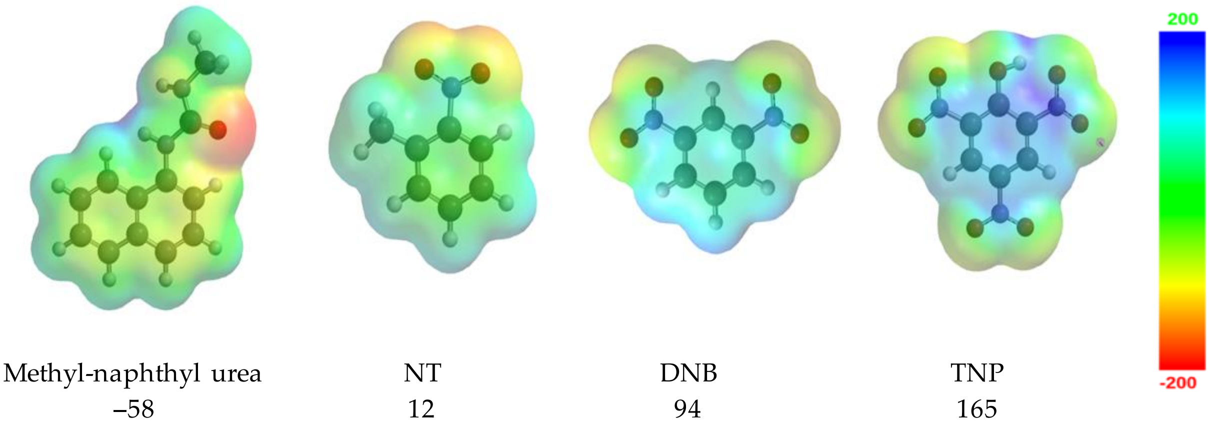

Figure S22. Electrostatic potential calculated by B3LYP/6-31G(d,p) (from red –200 kJ to blue +200 kJ). Mean value from the center of the aromatic ring (in kJ).
